# Oligoclonal Bands in Multiple System Atrophy: Case Report and Proposed Mechanisms of Immunogenicity

**DOI:** 10.3389/fnins.2022.852939

**Published:** 2022-02-28

**Authors:** Lee E. Neilson, Christopher Hollen, Amie Hiller, Lindsey Wooliscroft

**Affiliations:** ^1^Department of Neurology, Veterans Affairs Medical Center, Portland, OR, United States; ^2^Department of Neurology, Oregon Health and Sciences University, Portland, OR, United States

**Keywords:** ataxia, multiple system atrophy, oligoclonal band, neurodegenerative disease, neuroinflammation

## Abstract

Multiple System Atrophy (MSA) is a neurodegenerative disease with heterogeneous manifestations and is therefore difficult to diagnose definitively. Because of this, oftentimes an extensive workup for mimickers is undertaken. We herein report a case where the history and cerebrospinal fluid (CSF) findings of oligoclonal bands suggested an inflammatory disorder. Immunomodulatory therapy failed to ameliorate symptoms or alter the trajectory of continued physical decline, prompting re-visitation of the diagnosis. Oligoclonal bands, while generally viewed as specific to multiple sclerosis or other inflammatory conditions, may be seen in other disease processes. Therefore, this finding should not exclude consideration of neurodegenerative disease.

## Introduction

Multiple System Atrophy (MSA) is a neurodegenerative disorder akin to Parkinson’s disease (PD) that also manifests with significant autonomic dysfunction, pyramidal tract signs, and cerebellar findings (Fanciulli and Wenning, 2015), and in certain cases, it responds well to levodopa therapy (Martin et al., 2021). Because of its variable presentation, there is often a considerable delay in diagnosis and misdiagnosis of MSA (Koga et al., 2015). Immunologic conditions are among the mimics, so lumbar puncture is often considered to evaluate for oligoclonal bands and other signs of inflammation (Kim et al., 2016). Oligoclonal bands (OCBs) are generated when lymphocytes migrate into the central nervous system and stimulate the synthesis and release of immunoglobulins into cerebrospinal fluid (CSF) (Thompson and Keir, 1990). The presence of OCBs may approach 100% in multiple sclerosis (MS) (Chu et al., 1983; Thompson and Keir, 1990), but the specificity drops when considering other neuroinflammatory diseases (Petzold, 2013). Limited reports suggest that some neurodegenerative disorders may demonstrate OCB positivity, but no cases to our knowledge have been reported in MSA (Jesse et al., 2011). Herein, we report a case of MSA masquerading as a demyelinating condition and propose a reconsideration of the diagnosis when typical immunomodulatory therapy fails to ameliorate symptoms.

## Case Presentation

**FIGURE 1:**
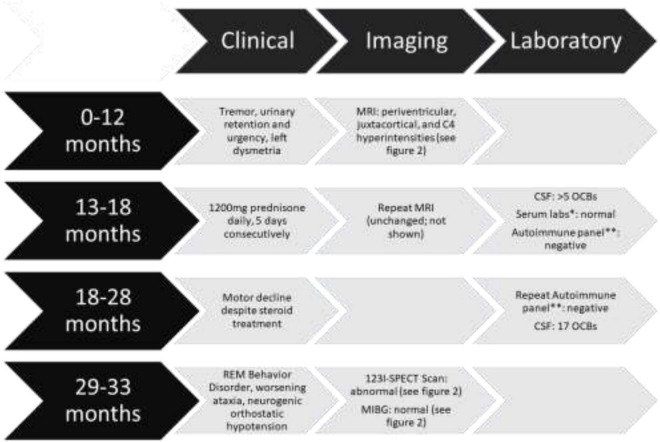
Timeline of events including clinical, imaging, laboratory findings and interventions *Serum labs included vitamin B1, B12, E, copper, zinc, thyroid-stimulating hormone (TSH), hepatitis B, antinuclear antibody (ANA), antineutrophil cytoplasmic antibody (ANCA), and tissue transglutaminase. All were reported within normal limits. **Autoimmune panel included: amphiphysin, AGNA-1, ANNA-1, ANNA-2, ANNA-3, CASPR2, CRMP-5, DPPX, GAD65, GRAF1, IgLONS, ITPR1, LGI1, mGluR1, NIF, NMDA, PCA-Tr, PCA-1, PCA-2 antibodies which were reported as “negative” except GAD65 which was reported as 0.00 nmol/L.

**FIGURE 2:**
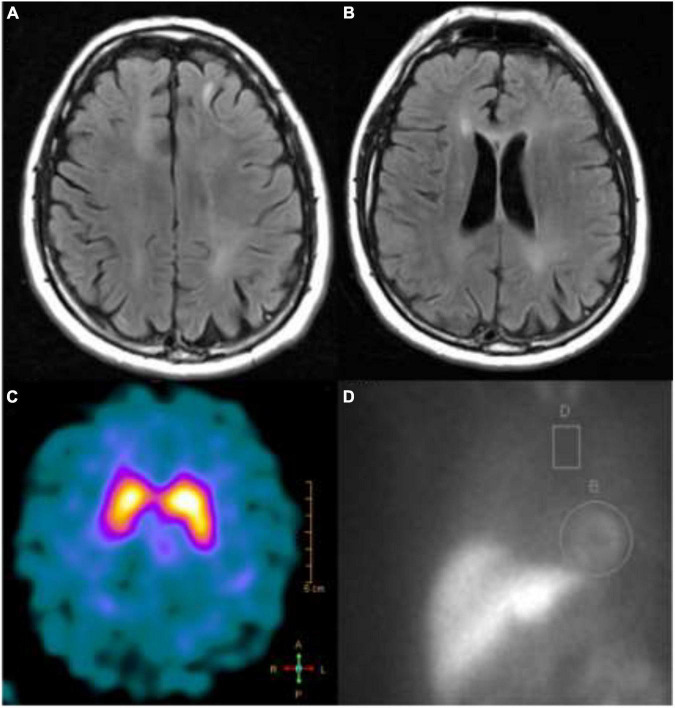
T2-FLAIR MRI showing **(A)** left frontal juxtacortical hyperintensity and **(B)** periventricular white matter hyperintensities; **(C)** a dopamine transporter scan showed reduced tracer uptake in the right putamen; **(D)** an MIBG scan showing higher than normal (>4:1) heart to mediastinum ratio.

A 56-year-old Caucasian male with a history of chronic obstructive pulmonary disease was initially referred for evaluation of bilateral upper extremity action tremor of 8 months duration (see Figure 1). The interview revealed gait instability with three falls in the preceding year, urinary retention and urgency, and navigational issues while driving. Initial examination revealed mild left-sided dysmetria, diffusely brisk reflexes and a bilateral upgoing plantar response. Magnetic resonance imaging (MRI) of the brain revealed periventricular white matter hyperintensities and a left frontal juxtacortical hyperintensity (see Figures 2A,B). No brainstem abnormalities were identified. MRI of the cervical spine revealed C5-C6 high-grade stenosis and a hyperintensity at C4.

Given the mixed exam findings, suspicious MRI lesions, and a second-degree relative with multiple sclerosis, a lumbar puncture was performed to evaluate for inflammatory causes of his symptoms. The initial CSF results yielded 3 white blood cells per mm^3^, 0 red blood cells per mm^3^, protein of 56 mg/dL, glucose of 65 mg/dL and > 5 OCBs restricted to CSF. He also had an elevated IgG index of 0.83 (normal 0.28–0.66) but normal IgG synthesis rate of 7.6 (normal ≤ 8.0 mg/d). It was at this point he was referred to neuro-immunology for further evaluation with a working diagnosis of primary progressive MS as he met the diagnostic criteria on the basis of 1-year of disability progression independent of clinical relapse, periventricular, and juxtacortical T2-hyperintense lesions, and positive CSF-specific OCBs.

A repeat MRI of the brain and cervical spine 6 months later showed no change in lesions and there were no incipient gadolinium-enhancing lesions. He did not meet diagnostic criteria for MS due to the lack of typical MS lesions. However, his abnormal CSF suggested an inflammatory process. The following additional lab results were normal or negative: vitamin B12, vitamin B1, vitamin E, copper, zinc, thyroid stimulating hormone, hepatitis B, ANA, ANCA, and tissue transglutaminase. A paraneoplastic panel from serum and CSF including amphiphysin, ANNA-1, CASPR2, CRMP5, DPPX, Gad65, IgLON5, LGI1, and NMDA were negative (see Figure 1). A screening computed tomography (CT) scan of the chest, abdomen, and pelvis was performed and no malignancy was identified.

He was treated empirically with high dose steroids (1,200 mg prednisone daily for 5 days consecutively). After steroid treatment, he continued to progress with more ataxia and increasing falls. He was then admitted for additional workup and rehabilitation assessment. A second lumbar puncture was performed (3 months after the first) which again identified 17 OCBs restricted to CSF. A second autoimmune panel from serum and CSF was sent with negative results. He was discharged to a rehab facility without any improvement following his hospitalization. On his return to our clinic, he demonstrated more marked ataxia and new emergence of suspected rapid eye movement behavior disorder (RBD). He was therefore referred to the movement disorder clinic for evaluation of a synucleinopathy. He denied symptomatic orthostasis, but his screening vitals did demonstrate a drop in systolic blood pressure of 32 mmHg. While he did not have pronounced parkinsonism, the cerebellar involvement, autonomic failure, upper motor neuron features, and RBD did suggest a diagnosis of MSA. 123I-Ioflupane single photon emission tomography (SPECT) imaging revealed reduced tracer uptake over the right putamen, indicative of nigrostriatal degeneration (see Figure 2C). Imaging of cardiac innervation with SPECT and [123I]metaiodobenzylguanidine (MIBG; see Figure 2D) demonstrated preserved sympathetic postganglionic neurons.

A revised diagnosis of MSA-cerebellar subtype was proposed. While he did not have classical findings on MRI of MSA, he met the clinically probable criteria (Gilman et al., 2008) and was counseled as such. He continues to slowly deteriorate but remains ambulatory with an assistive device (VIDEO).

## Discussion

CSF analysis is an important tool for the neurologist, and is considered an essential diagnostic test by the World Health Organization (2019). CSF is an ultrafiltrate and contains immunoglobulins passively transferred from the plasma, but since the original description of an abnormal fraction restricted to the CSF in the 1960s (Lowenthal et al., 1960), OCBs have become an important marker of inflammatory nervous system disease. The most recently revised McDonalds criteria re-emphasizes the importance of OCBs to establish dissemination in time; and for a diagnosis of primary progressive MS in the absence of lesions in the spinal cord, the presence of OCBs is required (Thompson et al., 2018). OCBs are also considered integral to the diagnosis of autoimmune encephalitis (Graus et al., 2016).

The proteins responsible for these bands are immunoglobulin G (IgG), and it is hypothesized they arise due to the activation of polyclonal B lymphocytes leading to synthesis of specific IgG molecules (Freedman et al., 2005). The gold standard technique to identify OCBs is isoelectric focusing on agarose gels followed by immunoblotting. This technique can identify one of five patterns: (1) No CSF-specific bands; (2) presence of CSF-restricted bands; (3) bands present in both compartments with some unique banding in CSF (as can be seen in systemic inflammatory diseases such as systemic lupus erythematosus); (4) bands present in both compartments indicating total passive transfer (as can be seen in chronic inflammatory polyradiculopathies; Ruiz et al., 2021); and (5) a monoclonal IgG pattern in both compartments, as can be seen myeloma (Deisenhammer et al., 2006). Because of this, it does require some technical expertise and experience, as there is inter-rater variability (Franciotta et al., 2005).

Previous studies evaluating the utility of OCBs in MS only compared the disease group with healthy controls; only more recently have laboratories used non-inflammatory neurological disease as a control. A large-scale analysis evaluated CSF in 765 patients with suspected neurodegenerative disease and identified only five patients with OCBs: one with motoneuron disease, one with a muscle disease, and three with Alzheimer’s disease. Zero patients with Parkinson’s disease or atypical parkinsonism had OCBs (Jesse et al., 2011). This recapitulated a much smaller study where zero of the five PD patients and zero of 13 Huntington disease patients demonstrated OCBs (Chu et al., 1983).

A more recent study evaluated 2,114 patients with non-inflammatory neurologic disease (Pannewitz-Makaj et al., 2020). They found that 22 patients (5.5%) of the 404 patients with neurodegenerative disease had >4 OCBs. It was not reported if any of these cases represented clinically probable MSA. While sample size may have influenced this surprising result, this group also employed a different technique of isoelectric focusing in polyacrylamide gels with consecutive silver staining. There is considerably reduced specificity for IgG and is therefore not considered routine clinical practice (Freedman et al., 2005).

While variability in technique may play some role, other hypotheses are certainly possible to explain the presence of OCBs in a presumed non-inflammatory, neurodegenerative disorder. This could be traced to a remote infection of the central nervous system (Jesse et al., 2011) or to an immune-mediated process in a known neurodegenerative disorder. It is becoming more well-established the synucleinopathies have underlying inflammatory components with both B-cells (Yang et al., 2020) and T-cells being implicated (Lindestam Arlehamn et al., 2020). This is perhaps unsurprising given the breakdown of the blood-brain barrier (Profaci et al., 2020). Even early MSA patients have been shown to have widespread microglial activation based on PET imaging (Kubler et al., 2019) and post-mortem analysis (Williams et al., 2020). This could imply that a peripherally-derived antigen stimulates local CNS synthesis of IgG proteins and not simply passive transfer. An alternative hypothesis comes from recent work showing that OCB target antigens are ubiquitous intracellular proteins, indicating that a B-cell response may partly be directed against these antigens during tissue destruction (Brandle et al., 2016). Whether this finding has prognostic value is unknown.

## Conclusion

CSF analysis, including determination of OCBs, is often essential in the workup of neurological disease. While the detection of OCBs generally indicates an inflammatory process is occurring, it should not, in isolation, lead to the exclusion of a non-inflammatory neurological disease, as its co-existence may be more common than realized.

## Data Availability Statement

The original contributions presented in the study are included in the article/supplementary material, further inquiries can be directed to the corresponding authors.

## Ethics Statement

Written informed consent was obtained from the individual(s) for the publication of any potentially identifiable images or data included in this article.

## Author Contributions

LN proposed the case report, analyzed the case, prepared the figures, and drafted the manuscript for intellectual content. LW, CH, and AH critically reviewed the manuscript. LN, LW, and CH were involved in the patients’ healthcare. All authors read and approved the submitted version.

## Conflict of Interest

The authors declare that the research was conducted in the absence of any commercial or financial relationships that could be construed as a potential conflict of interest.

## Publisher’s Note

All claims expressed in this article are solely those of the authors and do not necessarily represent those of their affiliated organizations, or those of the publisher, the editors and the reviewers. Any product that may be evaluated in this article, or claim that may be made by its manufacturer, is not guaranteed or endorsed by the publisher.
